# Performances of survival, feeding behavior, and gene expression in aphids reveal their different fitness to host alteration

**DOI:** 10.1038/srep19344

**Published:** 2016-01-13

**Authors:** Hong Lu, Pengcheng Yang, Yongyu Xu, Lan Luo, Junjie Zhu, Na Cui, Le Kang, Feng Cui

**Affiliations:** 1 State Key Laboratory of Integrated Management of Pest Insects & Rodents, Institute of Zoology, Chinese Academy of Sciences, Beijing, China; 2 Plant Protection College, Shandong Agricultural University, Tai’an, Shandong, China; 3 Beijing Institutes of Life Science, Chinese Academy of Sciences, Beijing, China

## Abstract

Insect populations feeding on different plant species are under selection pressure to adapt to these differences. A study integrating elements of the ecology, behavior, and gene expression of aphids on different host plants has not yet been well-explored. The present study explores the relationship between host fitness and survival, feeding behavior, and salivary gland gene expression of a pea (*Pisum sativum*) host race of *Acyrthosiphon pisum* feeding on a common host *Vicia faba* and on three genetically-related hosts (*Vicia villosa*, *Medicago truncatula*, and *Medicago sativa*). Life table data indicated that aphids on non-favored hosts exhibited small size, low reproduction rate, slow population increase and individual development, and long lifespan. Electrical penetration graph results showed that the aphids spent significantly less time in passive ingestion of phloem sap on all non-preferred host plants before acclimation. After a period of acclimation on *M. truncatula* and *V. villosa*, pea host race individuals showed improved feeding behavior. No individuals of the pea host race completed its life history on *M. sativa*. Interestingly, the number of host-specific differentially-expressed salivary gland genes was negatively correlated with the fitness of aphids on this host plant. This study provided important cues in host plant specialization in aphids.

Phytophagous insects frequently feed on a number of host-plant species with varying nutritional composition, chemical defenses, and physical texture^1^,^2^. Insect populations feeding on different plant species are under selection pressure to adapt to these differences, leading to the evolution of specialized host-adapted populations^3^. In the case of aphids, host plants exert considerable selection pressure because the plants constitute their feeding, mating, and oviposition sites. Therefore, host specialization in aphids evolves through the selection of behavioral and chemical mechanisms of host-plant recognition, as well as through metabolic adaptation to the phloem content of the host plant^4^. Unlike chewing insects, aphids obtain nutrients directly from the phloem through an intercellular route using their stylets, which are specialized mouthparts that do not cause major damage to plant tissues^5^,^6^. Exploring the relationship between fitness and feeding behavior of aphids in different host plants is an important issue of evolutionary biology.

Depending on the breadth of their host range, aphids have been classified as monophagous, oligophagous, polyphagous, and even pantophagous^7^. The pea aphid *Acyrthosiphon pisum* is a polyphagous species whose host-plant range covers dozens of plant genera of the legume family Fabaceae^8^,^9^,^10^,^11^. Many host-specific phytophagous insect species had arisen through shifting and adapting to new plants, especially in sympatric speciation. The pea aphid, like many other aphid species, is prone to forming sympatric populations showing differential preference and fitness on specific host plants^8^,^9^,^12^,^13^. Unusually for aphids, this genetic differentiation among host-adapted pea aphid genotypes has generated at least 11 well-distinguished sympatric host races in Western Europe, i.e., “A-K” biotypes^11^. Of them, “G” and “K” biotypes are named for the aphid population on pea (including *Pisum sativum* and *Vicia faba*) and on alfalfa (*Medicago sativa*), respectively^11^, although the cultivated broad bean, *V. faba*, is an universal host for all pea aphid genotypes^14^. In North America, pea aphid populations feeding on two important legumes, alfalfa (*M. sativa*) and red clover (*Trifolium pratense*), are highly specialized and reproductively isolated^13^,^15^. The availability of the genome sequence^16^ presents an enormous potential for the pea aphid as a model for understanding the genetic basis of host utilization.

Aphids feed on the phloem sap of host plants through stylet penetration, during which saliva is secreted from the aphid salivary gland, delivering effector proteins to overcome plant defenses^6^,^17^,^18^. Watery saliva is composed of a more complex mixture of enzymes and other components, which highly differ between aphid species and within the same species based on their diet^18^,^19^,^20^,^21^,^22^,^23^,^24^,^25^,^26^,^27^,^28^,^29^. These studies imply that the aphid adaptation range of host plants is largely linked to the variations of watery saliva composition. However, only few studies explored the relationship between feeding behavior and the salivary gland-expressed genes during aphid host alteration.

In this study, we aim to examine the degree to which a pea aphid host race retains the plasticity to “adapt” to other host plants within the broader host range of the species as a whole, using a single clone (YYC) of the “G” biotype collected from *P. sativum*. Ecologically, it is often the case that an agricultural landscape is dominated by a single aphid clone (the “superclone” phenomenon). So the question of the level of plasticity in a single clone is ecologically relevant. We maintain the clone in the laboratory on the neutral host *V. faba*, then compare its survival, feeding behavior, and salivary gland gene expression on three additional plants (*V. villosa*, *Medicago truncatula*, and *M. sativa*) representing preferred hosts for closely related host races^11^. We measure the performance of the YYC clone on each host plant before any acclimation to the new host has occurred, and again once the clone has had the opportunity to “adapt” to the new host plant.

## Results

### Demography of pea aphids on different host plants

**Figure 1:**
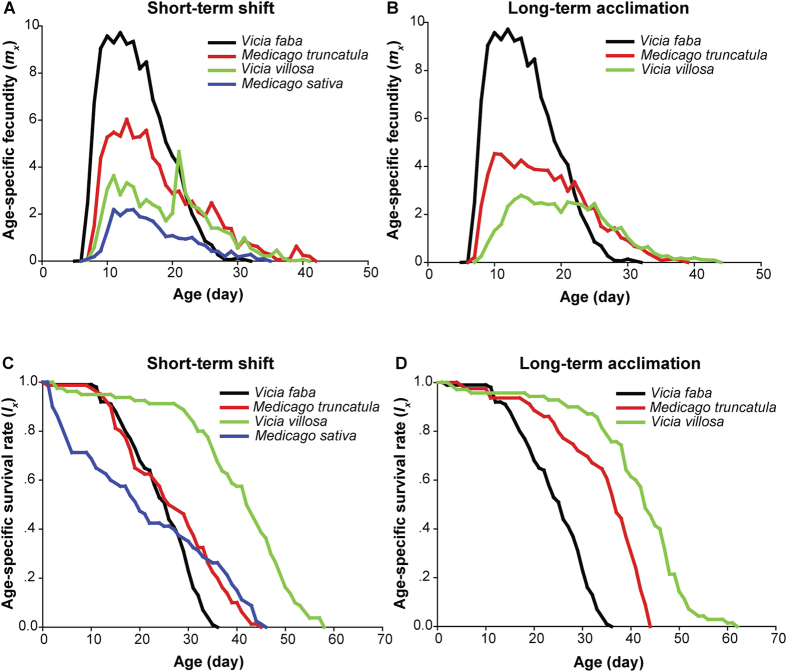
Age-specific fecundity (A,B) and survival rate (C,D) when the YYC clone was short-term shifted or long-term acclimation on *Vicia villosa*, *Medicago truncatula*, or *Medicago sativa*.

The non-acclimation life table reflects the initial response of aphids to a new host plant. For the life table experiment under non-acclimation state, the YYC clone was transferred to *V. villosa*, *M. truncatula*, or *M. sativa* within one generation (short-term shift). Remarkable decreases in the net reproductive rate, intrinsic rate of increase, and finite rate of increase were observed during the short-term shift to the three host plants, especially in *M. sativa*, on which the YYC clone showed the lowest net reproductive rate (*R*_0_ = 14 offspring), intrinsic rate of increase (*r*_*m*_ = 0.17 d^−1^), and finite rate of increase (*λ* = 1.19 d^−1^) (Table 1). Two reproductive peaks appeared when the YYC clone was transferred to *V. villosa* (Fig. 1A). By contrast, the mean generation time and the period of nymph and adult were significantly increased during the short-term shift (Table 1), but a high nymphal mortality was observed when the YYC clone was transferred to *M. sativa* (Fig. 1B).

**Figure 2:**
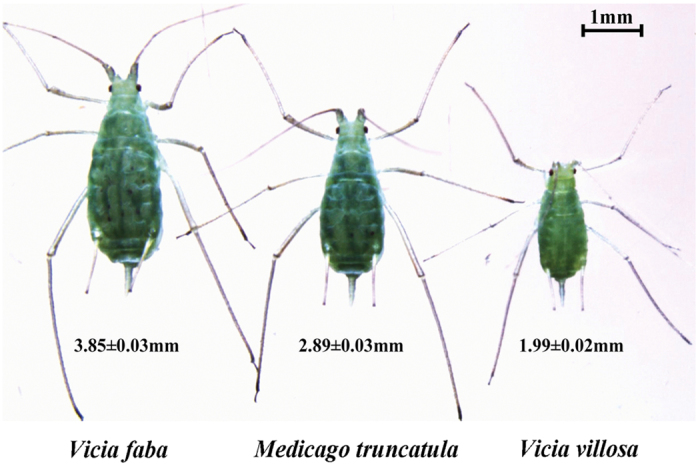
Body sizes of adult pea aphids adapted to different host plants. The YYC clone was transferred to *Vicia villosa*, *Medicago truncatula*, or *Medicago sativa* for six months of acclimation. Stable colonies were established on *V. villosa* and *M. truncatula*, but not on *M. sativa*. The average body length (from vertex to cauda, 30 individuals) of each stable colony was measured (mean ± SE).

For the life table experiment following acclimation, the YYC clone was transferred to *V. villosa*, *M. truncatula*, or *M*. *sativa* for long-term acclimation. After six months, stable colonies were established on *V. villosa* and *M. truncatula*, but not on *M. sativa*. This means that host specialization of the pea aphid was not overcome when transferring from *V. faba* to *M. sativa*. The life histories were compared among *V. faba*, *V. villosa*, and *M. truncatula* colonies to evaluate aphid alteration to different host plants after a long-term acclimation. The YYC clone had the highest net reproductive rate (*R*_0_ = 101 offspring), intrinsic rate of increase (*r*_*m*_ = 0.38 d^−1^), and finite rate of increase (*λ* = 1.46 d^−1^), followed by the *M. truncatula* colony (*R*_0_ = 65 offspring; *r*_*m*_ = 0.29 d^−1^; *λ* = 1.34 d^−1^), and then the *V. villosa* colony (*R*_0_ = 46 offspring; *r*_*m*_ = 0.22 d^−1^; *λ* = 1.25 d^−1^) (Table 1). The *M. truncatula* and *V. villosa* colonies exhibited flatter reproductive peaks (i.e., a longer overall duration of reproduction) than that of the YYC clone (Fig. 1C). The mean generation time was shortest in the YYC clone (*T* = 12 d), followed by the *M. truncatula* colony (*T* = 14 d), and longest in the *V. villosa* colony (T = 17 d) (Table 1). The YYC clone had the shortest longevity (24 d), whereas the *V. villosa* colony had the longest longevity (41 d), indicating a difference of 1.7-fold. Such a large difference was mainly attributed to the difference in adult longevity (1.9-fold) (Table 1, Fig. 1D). The body sizes of aphids varied when aphids fed on different host plants. Taking the adults as an example, the average body length of YYC clone was the largest (3.85 ± 0.03 mm), followed by *M. truncatula* colony (2.89 ± 0.03 mm), and the smallest was *V. villosa* colony (1.99 ± 0.02 mm) (Fig. 2).

In brief, the demographic results indicated that the fitness of pea aphids was highest in *V. faba*, followed by *M. truncatula*, *V. villosa*, and finally *M. sativa*.

### Feeding behavior of pea aphids on different host plants

**Figure 3:**
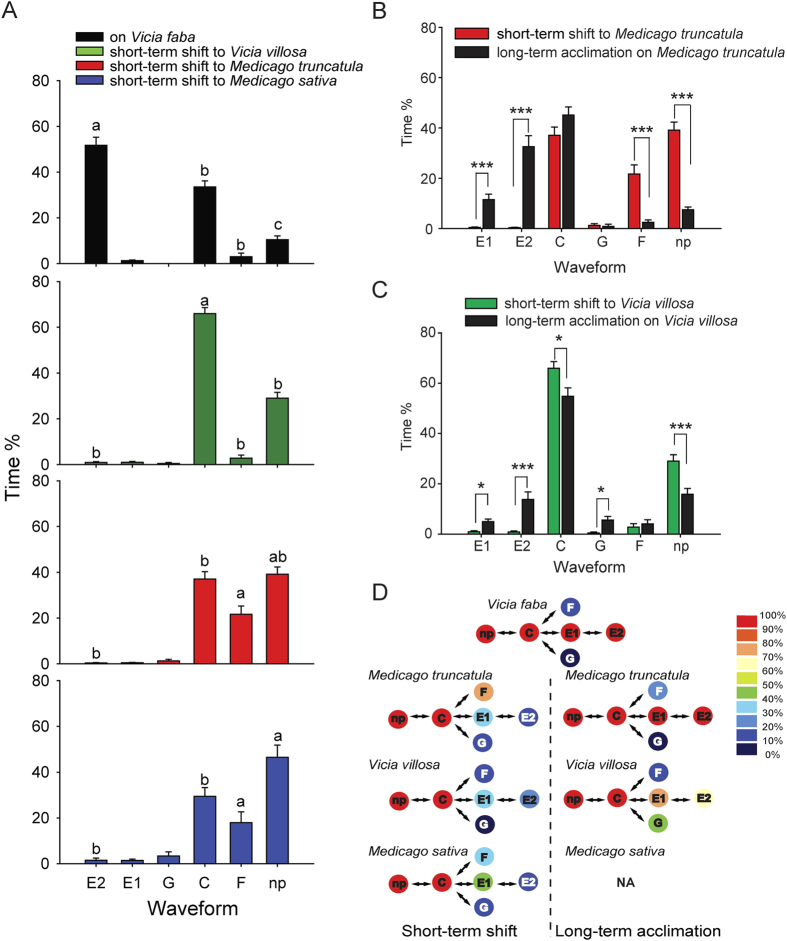
Feeding behaviors of pea aphids fed on different host plants measured by electrical penetration graph technique. E1, watery salivation. E2, passive ingestion. C, probing plant cells. F, derailed stylet. G, drinking from xylem. np, non-probing. Time ratios (time spent in each waveform divided by 5 h) were reported as mean ± SEM. (**A**) Comparison of feeding behavior of the YYC clone before and after a short-term shift to *Medicago truncatula*, *Vicia villosa*, or *Medicago sativa*. Different letters above columns indicates a significant difference of the same waveform in different groups evaluated by ANOVA using SPSS 17.0. (**B**) Comparison of feeding behavior of the YYC clone after a short-term shift to *M. truncatula* for 5 h and after a long-term acclimation on *M. truncatula* for six months. ***P < 0.001. (**C**) Comparison of feeding behavior of the YYC clone after a short-term shift to *V. villosa* for 5 h and after a long-term acclimation on *V. villosa* for six months. *P < 0.05; ***P < 0.001. (**D**) Model diagram to show feeding behavior patterns of the pea aphids before and after alteration to different host plants. Colors represent the ratios of individuals entering a certain feeding step in a group of aphids (30 individuals per group).

To monitor the feeding behavioral performance of the aphids on different host plants, the electrical penetration graph (EPG) technique (Tjallingii 1988) was employed to display the feeding behavioral traits. When the YYC clone was directly transferred to the other three plants, the time spent in passive ingestion of phloem sap (E2 wave) decreased dramatically, and the non-probing (np wave) time increased significantly (Fig. 3A). In addition, when feeding on *V. villosa*, the YYC clone spent more time probing plant cells to seek phloem sap (C waves) (Fig. 3A). On the two *Medicago* plant species, the YYC clone met penetration difficulties owing to derailed stylet mechanics, as shown by a higher ratio of F wave time (Fig. 3A). The colonies were characterized by different feeding performances when the feeding behavior of the colonies (30 individuals per group) were taken as a whole (Fig. 3D). For the YYC clone, 100% of the individuals went through the main feeding steps, from non-probing (np), to probing plant cells (C), to watery salivation (E1), and finally to passive ingestion of phloem sap (E2). When the YYC clone was transferred to the other three host plants, the feeding behavior of most individuals stopped at the probing step, and phloem sap ingestion was retarded. Around 80% and 40% of individuals had penetration difficulties (F) on *M. truncatula* and *M. sativa*, respectively.

After acclimation on *M. truncatula* or *V. villosa* for six months, the feeding behavior of the aphids improved (Fig. 3B,C). The time of watery salivation (E1 wave) and passive ingestion of phloem sap (E2 wave) increased, and the non-probing (np wave) time decreased significantly in both *M. truncatula* and *V. villosa* colony. The penetration difficulties (F wave) were lessened remarkably in the *M. truncatula* colony (Fig. 3B). Surprisingly, the *V. villosa* colony spent more time in drinking water from xylem (G wave) compared with the non-acclimation group (Fig. 3C). The three colonies were characterized by different feeding performances through the above same method (Fig. 3D). The stable *M. truncatula* colony showed a feeding pattern quite similar to the YYC clone. The feeding behavior of the *V. villosa* colony was also improved, as 70–80% individuals were able to enter the ingestion step (E1 and E2), and around 50% individuals ingested xylem contents (G). The YYC clone did not adapt to *M. sativa* from the outset, probably caused by failure to overcome penetration difficulties.

**Figure 4:**
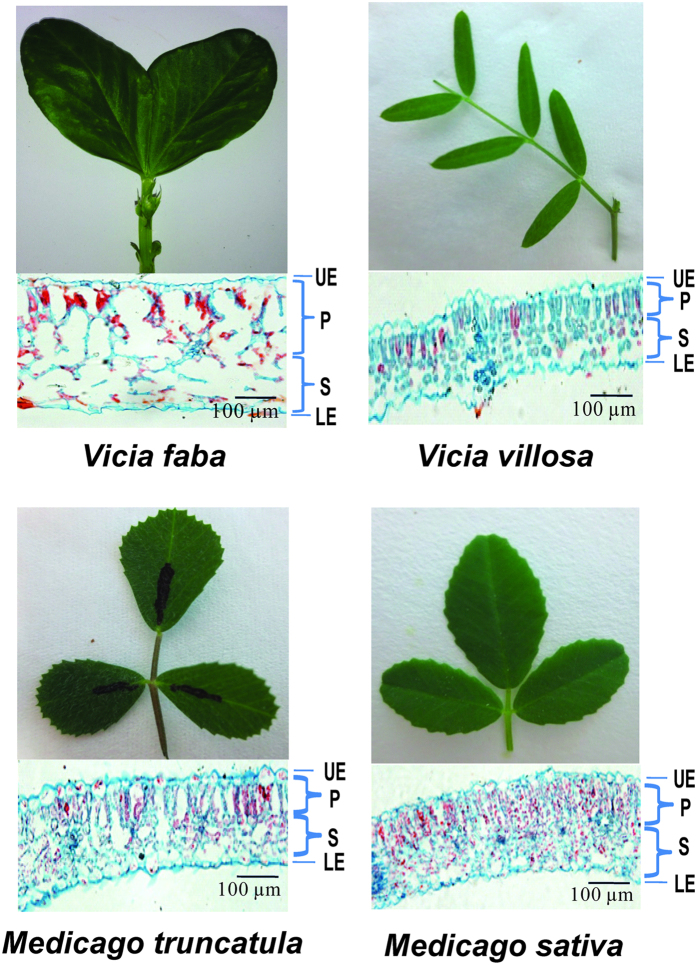
The leaf microstructure of *Vicia faba*, *Vicia villosa*, *Medicago truncatula*, and *Medicago sativa*. Cross sections of leaves were stained in 0.05% aqueous safranin and counterstained with an alcoholic solution of fast green FCF. P, palisade tissue. S, spongy tissue. UE, upper epidermis. LE, lower epidermis.

The differences in feeding behavior among aphids on different host plants could be partially related to the leaf microstructures. Among the four plants, the cells of *V*. *faba* were largest, whereas the cells of *V*. *villosa* were smallest; the cells of *V*. *villosa* were arranged most loosely, whereas the cells of the two *Medicago* plants were arranged most closely, especially the palisade tissue cells of *M*. *sativa* (Fig. 4).

### Transcriptomic characteristics of pea aphid salivary glands

To reveal the relationships between feeding behavior and gene expression, we conducted transcriptomic analysis in the salivary glands of pea aphids from the YYC clone, *V. villosa* and *M. truncatula* colonies, as well as from the YYC clone feeding on *V. villosa*, *M. truncatula*, or *M. sativa* for 5 h. At least 10 million of 49 bp high quality RNA-Seq reads were obtained for each sample with Q20 larger than 99% and Q30 larger than 96%. Around 12,000 genes of pea aphids were detected for expression in each sample, and 16,712 genes were expressed in aphid salivary glands, 1,333 of which were predicted to encode secretory proteins (Data S1, Supporting Information). For the salivary gland-expressed genes, the significantly enriched molecular functions (Level 3 of GO terms) were small molecule binding, lipid binding, carbohydrate binding, hydrolase activity, oxidoreductase activity, ligase activity, enzyme activator activity, nucleoside-triphosphatase regulator activity, substrate-specific transporter activity, transmembrane transporter activity, and structural constituent of ribosome (Data S2, Supporting Information). The significantly enriched molecular functions for secretory protein encoding genes were carbohydrate binding, hydrolase activity, peroxidase activity, and pigment binding (Data S2, Supporting Information).

### Differentially expressed genes of pea aphids while shifting to different host plants

**Figure 5:**
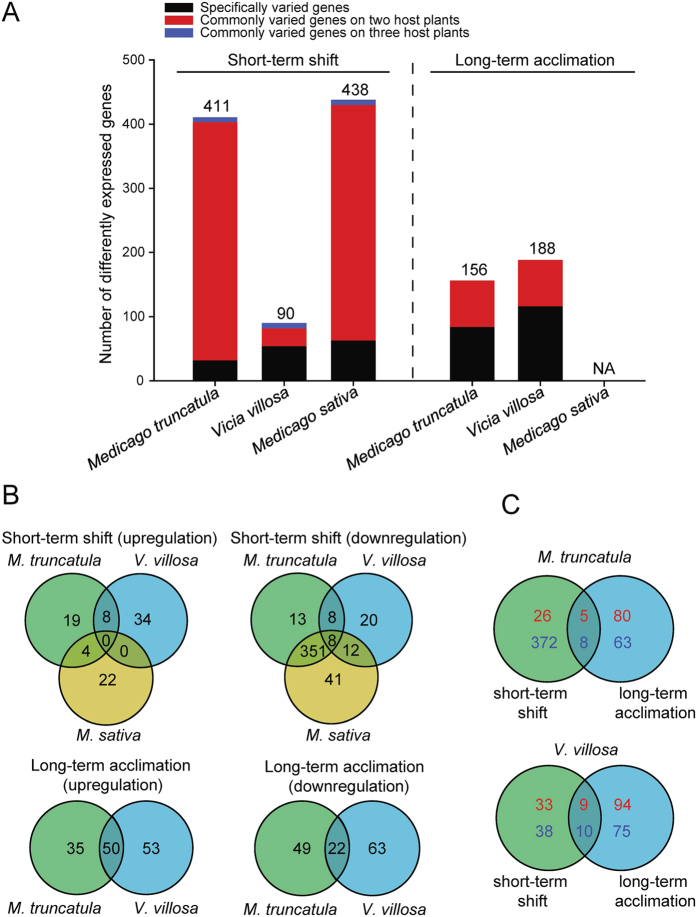
Numbers of differently expressed genes in aphid salivary glands when the YYC clone was short-term shifted to or long-term acclimation for six months on *Vicia villosa*, *Medicago truncatula*, or *Medicago sativa*. (**A**) Total numbers of differentially expressed genes. (**B**,**C**) Venn diagram showing the similarity of differentially expressed genes. Red and blue numbers in (**C**) delegate upregulated and downregulated gene numbers, respectively.

Gene expression in salivary glands of the YYC clone was compared before and after this clone was transferred to *M. truncatula*, *M. sativa*, or *V. villosa* for feeding 5 h. The expression levels of 411, 438, and 90 genes were changed, respectively, and 30 genes were predicted to encode secretory proteins (Fig. 5A, Data S3, Supporting Information). The degree of gene expression change when the YYC clone was transferred to the two *Medicago* plants was more similar than when the YYC clone was transferred to *V. villosa* (Fig. 5A). The number of genes that varied in aphids for specific host plant was 32 on *M. truncatula*, 54 on *V. villosa*, and 63 on *M. sativa* (Fig. 5B), in which the fitness of the aphids were negatively correlated with such order of these host plants.

No gene was commonly upregulated, but 8 genes were commonly downregulated during the short-term shift from broad bean to other three host plants (Fig. 5B). The commonly downregulated genes included 5 heat shock proteins (Data S4, Supporting Information). Among the 32 genes that varied specifically on *M. truncatula*, the top upregulated genes were P450 6k1, alkaline phosphatase, and three unknown function genes. The top downregulated genes included four genes of unknown function, a mitochondrial coenzyme A transporter, and a cyclin-dependent kinase regulatory subunit (Data S3, Supporting Information). Of the 54 genes that varied specifically on *V. villosa*, actin-related protein 10, nucleolin, and splicing factor 3A subunit 1 were strongly upregulated, and one takeout protein and two chemosensory proteins were moderately upregulated. Lysozyme, vav protein, COP9 signalosome complex subunit 1b, and RNA-directed DNA polymerase were greatly downregulated (Data S3, Supporting Information). Of the 63 genes that varied specifically on *M. sativa*, fasciclin-2 was the top upregulated gene in addition to four function unknown genes. MOB kinase activator-like 1, apoptosis-antagonizing transcription factors (AATFs), furin-like protease 2, lava lamp protein, short-chain dehydrogenase/reductase, and oligoribonuclease were greatly downregulated (Data S3, Supporting Information).

### Differentially expressed genes in three pea aphid colonies

Gene expressions in salivary glands of the *M. truncatula* and the *V. villosa* acclimation colonies were compared with those of the YYC clone. A total of 156 genes and 188 genes were differentially expressed in the *M. truncatula* and *V. villosa* colonies, respectively, and 47 genes putatively encoded secretory proteins (Fig. 5A, Data S5, Supporting Information). The commonly varied genes in the two colonies numbered 72, including 50 upregulated genes and 22 downregulated genes (Fig. 5B). The number of genes that varied in a specific colony was 84 in the *M. truncatula* colony and 116 in the *V. villosa* colony (Fig. 5B), which was negatively correlated with the fitness of the aphids on these host plants.

Among the 50 commonly upregulated genes, only 5 had annotations, namely, alpha-(1,6)-fucosyltransferase, acyl-CoA-binding protein, calpain-7, transcription elongation factor, and syntaxin. Of the 22 commonly downregulated genes, three takeout genes were largely downregulated (Data S4, Supporting Information). The specifically upregulated genes in the *M. truncatula* colony were 35, of which inositol-trisphosphate 3-kinase was the most upregulated gene. The specifically downregulated genes in the *M. truncatula* colony were 49, top of which were five function unknown genes and furin-like protease 2 (Data S5, Supporting Information). The specifically upregulated genes in the *V. villosa* colony were 53, among which two cathepsin Bs and one takeout protein were moderately upregulated. The specifically downregulated genes in the *V. villosa* colony were 63, among which five function unknown genes, inositol-tetrakisphosphate 1-kinase, and MOB kinase activator-like 1 were largely downregulated (Data S5, Supporting Information).

There were few genes in common between those differentially-expressed after the short- and long-term host acclimation periods. A small number of genes, i.e., 13 on *M. truncatula* and 19 on *V. villosa*, commonly varied during the short-term shift and long-term acclimation, while most genes (over 80%) varied only under one state (Fig. 5C). Stress-related genes, such as seven heat shock proteins and stress-induced-phosphoprotein 1, were only involved in short-term shift.

## Discussion

Aphids are important organisms in the study of host specialization and ecological speciation in animals. Pea aphid is quite unique amongst aphids in forming genetically-distinct host races, due in part to its lack of host alternation. Pea aphid can complete its full life cycle on a single plant host, many of which are now grown in large monocultures, leading to the development of sympatric host races over the past 10,000 years^30^. In this study, we examined the survival, feeding behavior, and salivary gland gene expression of pea aphids on a rearing host and three additional Fabaceae plant species before and after a period of acclimation of at least 6 months. Because rearing host is known to be a factor influencing future performance, we chose to rear our clone on a “neutral” host plant that is suitable for all pea aphid host races, *V. faba*^14^. We have focused our study on a single clone (YYC) in order to assess the level of host use plasticity, rather than genetic variation, that is retained in the pea aphid host races. In doing so, we have found that after thousands of years of adaptation towards feeding on a single host (*P. sativum*), the “G” host race of pea aphid retains some plasticity to feed on plants that successfully host other races – albeit with significantly reduced performance. After at least 6 months of acclimation, the clone YYC we tested in our experiments was able to feed and reproduce successfully on two alternate hosts (*V. villosa, M. truncatula*), but not on *M. sativa*. Of the alternate hosts, the clone YYC performed best across all life history characters on *M. truncatula* compared to *V. villosa*.

Our results confirm that pea aphid host races have undergone significant host specialization. Previous genetic analyses of both the aphid^11^ and its primary symbiont^30^ have indicated that the host races adapted to *Vicia* spp. and *Medicago* spp. have diverged most recently (~1000 years) from the pea host race used in this study^30^. Hence, one might expect that of all the alternate hosts, *Vicia* spp. and *Medicago* spp. would be most likely to remain suitable.

EPG analysis clearly showed that aphid performance on different hosts was related to the capacity of the aphid to feed successfully on phloem sap in each plant (E2). After 6 months of acclimation, the clone YYC was not able to achieve phloem ingestion on *M. sativa*, but could do so on *M. truncatula* and, to a significantly lesser degree, on *V. villosa*. These results contrast with earlier studies, which showed that differences in the feeding behavior of pea aphid host races from *Medicago*, *Pisum*, and *Trifolium* were mainly due to the incidence of electric potential drops that are indicative of brief punctures of plant cells and the duration of salivation (E1 wave)^31^. Feeding behavior is a good indicator of host acceptance for aphids. Aphids do not distinguish between hosts and non-hosts at a distance; rather, they determine whether the plant is suitable or not after the first insertion of the stylet in the cuticle and the epidermis of the leaf^13^,^32^. Host acceptance or rejection essentially depends on the successful completion of successive feeding behaviors. Numerous plant factors influence aphid host selection, including attractive or repellent volatiles^33^,^34^, deterrent epicuticular lipids^35^, glandular trichomes^36^, deterrent gustatory cues in the epidermis^37^, or compounds inhibiting stylet penetration in the mesophyll^36^. Perhaps the most important plant factors influencing aphid feeding behavior are located in the epidermis and sieve elements^38^.

Our results suggest that the YYC clone may have encountered mechanical obstacles from the closely arranged palisade tissue cells of the two *Medicago* plants during feeding. However, this does not explain why the YYC performed quite differently on the two *Medicago* plants. Flavonoid glycosides in *M. sativa* affect the feeding behavior of pea aphids^39^. Feeding on the isoflavone genistein and the flavone luteolin prolonged the period of stylet activity (C wave), reduced salivation (E1 wave), and passive ingestion (E2 wave) in pea aphids; at higher concentrations (≥100 μg cm^−3^ for luteolin, ≥1,000 μg cm^−3^ for genistein), the flavonoids completely stopped these activities^40^. Whether *M. truncatula* contains lower levels of flavonoid glycosides than *M. sativa* requires further investigation.

The YYC clone was not able to feed effectively on any of the alternative host plants in the first generations after transfer. Long bouts of phloem feeding (E2) behaviors were only observed after a long period of acclimation to either *V. villosa* and *M. truncatula*. Host plant acclimation is often addressed in experimental designs examining aphid-plant interactions^8^,^9^,^13^,^15^,^41^, but there is little information on the underlying mechanisms. Our results indicate that the YYC clone could only achieve limited phloem feeding during this acclimation period to new hosts, irrespective of whether successful feeding eventuated after the acclimation period. Because salivary proteins have an important role in mediating host plant interactions, we examined salivary gland gene expression for insights into the host acclimation process. Interestingly, the short term transcriptional response was not indicative of future successful acclimation. The similarity in expression pattern (*M. truncatula* vs *M. sativa*; *V. faba* vs *V. villosa*) was correlated to host plant relatedness more than eventual successful performance. Rather, the largest differences in gene expression were observed on the two *Medicago* spp., including a high proportion of shared downregulated genes. This shared response could be indicative of shared chemistry between these two congeneric hosts, or it may be a direct response to the mechanical feeding difficulties (F wave) observed upon transfer to these hosts from *V. faba*. The eventual successful performance was negatively correlated with the number of host-specific differentially-expressed salivary gland genes.

The immediate response was more indicative of the stress of a non-congeneric host switch. We found that the stress-relative genes, such as seven heat shock proteins and stress-induced-phosphoprotein 1, were only involved in the short-term shift, but these genes were downregulated. Heat shock proteins are a family of molecular chaperones that are generally upregulated in response to environmental stresses, including starvation^42^. In *Aphis glycines*, nine heat shock proteins were upregulated when the aphids fed on resistant soybean compared with susceptible soybean^43^. Stress-induced-phosphoprotein 1 is known as heat shock protein-organizing protein and acts primarily as an adapter to modulate the chaperone activities of heat shock proteins^44^.

Amongst the differentially expressed genes during host alteration, we identified 60 secretory protein-encoding genes, of which only 29 genes have annotations. Of the 42 most abundant secreted salivary gland proteins^18^, only two, ACYPI005818 (no annotation) and ACYPI004198 (apolipophorin), changed expression levels in the host alteration process. Secreted apolipophorins interfere with signals of plant cellular immune response by binding to lipid elicitor molecules^45^. In addition to ACYPI004198, two other apolipophorins (ACYPI36683, ACYPI008569) were downregulated when pea aphids adapted to unfavorable plants, probably to avoid the plant’s immune response. No functionally characterized pea aphid saliva proteins, such as C002^46^, Armet^28^, angiotensin-converting enzyme^21^,^29^, M1 zinc-dependent metalloprotease, glucose-methanol-choline-oxidoreductase, and regucalcin^21^ showed differential expression during host alteration.

In conclusion, our study reveals the plasticity involved in aphid-host plant alteration from demography and feeding behavior to the standpoint of gene expression in salivary glands. The new knowledge from this study will further our understanding of the mechanisms generating host-plant specialization and lead to improved management strategies of piercing-sucking pest insects. On the other hand, although many genes are found differentially expressed in salivary glands, the possible functions of these differentially expressed genes relative to adaptation plasticity need further studies in future. Conducting equivalent studies on clones representing other host races would be a very interesting follow-up.

## Materials and Methods

### Insects and host plants

A field population of pea aphid was collected from *P. sativum* (G biotype) in 2010 and has been reared on *V. faba* at lab as an experimental colony YYC since then^28^. Many aphids of the YYC colony were transferred to *V. villosa*, *M. truncatula* (A17 line), or *M. sativa* for rearing for at least six months. However, stable colonies were only established on *V. villosa* and *M. truncatula*, but not on *M. sativa*. These three colonies were raised in environmental chambers at 21 ± 1 °C, a photoperiod of 16 h:8 h (light:dark), and 60% ± 5% relative humidity. The adult body length of each colony was measured from vertex to cauda, and photos of the bodies were taken under a stereomicroscope (Leica, Wetzlar, Germany). Thirty aphids were measured for each colony and the body length was recorded as mean ± SEM.

### Life table construction

A life table displays the demographic characteristics of a population. Life tables were constructed in two categories of experiments. The first one was to study the survival of the YYC clone transferred to *V. villosa*, *M. truncatula*, and *M. sativa* for one generation (i.e., non-acclimation state). The second one was to study the survival of three stable aphid colonies on three distinct host plants *V. faba*, *V. villosa*, and *M. truncatula* (i.e., acclimation state). For the first experiments, 80 first instar nymphs of the YYC clone were collected within 12 h of birth and were raised individually on *V. villosa*, *M. truncatula*, or *M. sativa* leaves. For the second experiments, 80 first instar nymphs of the YYC clone, *V. villosa* or *M. truncatula* colony were collected within 12 h of birth and were raised individually on their respective host plant leaves. Single individual of first instar nymphs was maintained on one leaf. The leaf stem was wrapped with wet cotton in a 3.5 cm-diameter Petri dish containing water. The leaf and the 3.5 cm-diameter Petri dish were put in a 9 cm-diameter Petri dish and covered with a layer of plastic membrane with small holes for aeration. The aphids were raised in the same environmental conditions as above. Survival, ecdysis, and number of newborn nymphs were recorded every day from birth to death for each aphid. Using cut leaves in life table study has been reported in many aphids^47^,^48^.

The raw data of the life table studies were analyzed using the TWOSEX-MSChart program^49^, based on the age-stage, two-sex life table theory^50^,^51^. Accordingly, the age-specific survival rate (*l*_*x*_) and age-specific fecundity (*m*_*x*_) were calculated (x = age in days). The intrinsic rate of increase (*r*) was estimated according to the iterative bisection method from the Euler-Lotka formula^52^. The finite rate of increase *λ* was calculated as *λ* = *e*^*r*^. The net reproductive rate (*R*_0_) was computed as the mean number of offspring that an individual can produce during its lifetime. The mean generation time (*T*) was defined as the time that a population requires to increase to *R*_0_-fold of the number at the stable age-stage distribution. The means and standard errors of population parameters were calculated by the bootstrap method^53^ using the TWOSEX-MSChart program.

### Electrical penetration graph (EPG) measurements

The EPG technique^54^ was employed to detect the feeding behaviors of the YYC clone, *V. villosa* and *M. truncatula* colonies on their respective plants, and the YYC clone short-term shifted to *V. villosa*, *M. truncatula*, or *M. sativa*. The intact plants of 2–3 week old *V. faba*, *V. villosa* and *M. sativa*, and 4–5 week old *M. truncatula* were used. For each group, 30 to 40 fourth instar nymphs were chosen for 5 h EPG waveform recordings using a Giga-8 DC EPG System (EPG Systems, Wageningen, Netherlands). Six waveforms, namely, E1, E2, C, F, G, and np, were analyzed with the software Stylet+ of the same company as described by^5^, and their time ratios were reported as mean ± SEM. Pairwise or multiple comparison was analyzed using the *t*-test or one-way ANOVA using SPSS 17.0.

### Leaf tissue microstructure

The leaf microstructures of the four host plants were observed using the method described by Gram and Jorgensen^55^. The fourth or fifth leaves from the top of stems of 2–3 week old *V. faba*, *V. villosa*, *M. sativa*, and 4–5 week old *M. truncatula* were collected and fixed for 12 h in a solution of 5 ml 40% formaldehyde, 5 ml glacial acetic acid, and 90 ml 50% ethanol. After dehydration through a gradient series of ethanol, the leaves were embedded in paraffin and sliced at a thickness of 15 μm. After deparaffinization, the sections were stained in 0.05% aqueous safranin for 2 h and counterstained with an alcoholic solution of fast green FCF (0.1 g fast green in 100 ml 95% ethanol) for 15 s. The sections were then observed and photographed using DMI4000B microscope (Leica, Wetzlar, Germany).

### RNA isolation from salivary glands

Salivary glands of pea aphid adults (female and unwinged) from the YYC clone, *V. villosa* and *M. truncatula* colonies and from the YYC clone after feeding on *V. villosa*, *M. truncatula*, or *M. sativa* for 5 h were dissected in 0.9% saline solution and put immediately in TRIzol reagent (Invitrogen, Carlsbad, CA, USA) at 0 °C. Total RNA was isolated from pooled 500 salivary glands of each colony using TRIzol according to the manufacturer’s instructions. Genomic DNA contamination was removed from RNA using TURBO DNA-free kit (Ambion, Austin, TX, USA).

### Transcriptome sequencing and analysis

Paired-end RNA-seq libraries were prepared following Illumina’s protocols and sequenced on the Illumina HiSeq^TM^ 2000 sequencer (Illumina, Inc., San Diego, CA, USA) with 49 base-pair (bp) single-end. At least 10 million reads were obtained for each sample and mapped to the pea aphid genome using TopHat2^56^. The genome sequence and gene annotation data sets were downloaded from AphidBase Official Gene Set v1 (http://www.aphidbase.com/). For genes with multiple isoforms, the longest isoform was selected as representative. Gene expression levels were measured using RPKM (reads per kb per million reads) method. Differentially expressed genes were detected based on the Poisson distribution as suggested by Audic & Claverie^57^. To reduce the various biases, we further used the trimmed mean of M-values (TMM) method^58^ to eliminate the influence of differences in RNA output size between samples. Bias of multiple testing was adjusted by Benjamini-Hochberg method^59^. Expression ratio ≥ 2 and adjusted *P* value < 0.001 were used as a threshold of significance of the differences in gene expression.

Functions of protein coding genes were assigned according to the best match derived from alignments to proteins in NR database using BLASTP with E-value < 1E-5. Motifs and domains were identified by searching against the InterPro database using InterProScan. Gene Ontology (GO) annotation was retrieved from the InterPro database. The presence of a signal peptide was detected using SignalP (http://www.cbs.dtu.dk/services/SignalP/). GO enrichment analysis for the supplied gene list was carried out using our in-house Perl/R scripts, which were constructed based on an algorithm presented by GOstat^60^ with the whole annotated gene set as the background. The Perl/R scripts can be downloaded from GitHub (https://github.com/pengchy/EACO). The *P* value was approximated using the chi-square test. Fisher’s exact test was applied when any expected value of count was below 5. The false discovery rate was calculated to adjust multiple testing using the Benjamini-Hochberg method^59^.

## Additional Information

**Accession codes:** Illumina sequence data for salivary glands of pea aphid adults from the YYC clone, *V. villosa* and *M. truncatula* colonies and from the YYC clone after feeding on *V. villosa*, *M. truncatula*, or *M. sativa* for 5 h have been deposited in the NCBI Sequence Read Archive (Accession no: SRP055790).

**How to cite this article**: Lu, H. *et al*. Performances of survival, feeding behavior, and gene expression in aphids reveal their different fitness to host alteration. *Sci. Rep*. **6**, 19344; doi: 10.1038/srep19344 (2016).

## Supplementary Material

Supplementary Information

Supplementary Dataset 1

Supplementary Dataset 2

Supplementary Dataset 3

Supplementary Dataset 4

Supplementary Dataset 5

## Figures and Tables

**Table 1:** Life-table parameters (mean ± SE) of the YYC clone after a short-term shift or a long-term acclimation on different host plants.

Parameters	*Vicia faba*	short-term shift			long-term acclimation	
		*Medicago truncatula*	*Vicia villosa*	*Medicago sativa*	*Medicago truncatula*	*Vicia villosa*
Net reproductive rate, R_0_ (offspring)	101.2 ± 3.7	62.6 ± 3.9	50.4 ± 2.2	14.3 ± 1.7	64.9 ± 2.7	46.0 ± 1.9
Intrinsic rate of increase, r_m_ (day^−1^)	0.38 ± 0.004	0.30 ± 0.005	0.25 ± 0.005	0.17 ± 0.009	0.29 ± 0.005	0.22 ± 0.005
Finite rate of increase, λ (day^−1^)	1.46 ± 0.006	1.35 ± 0.007	1.28 ± 0.006	1.19 ± 0.011	1.34 ± 0.006	1.25 ± 0.007
Mean generation time, T (day)	12.3 ± 0.1	13.8 ± 0.2	15.6 ± 0.2	15.3 ± 0.3	14.3 ± 0.2	17.3 ± 0.4
Nymph period (day)	7.4 ± 0.08	8.2 ± 0.09	8.6 ± 0.12	8.9 ± 0.15	7.9 ± 0.10	10.1 ± 0.22
Adult period (day)	17.2 ± 0.68	18.7 ± 1.10	33.6 ± 1.01	20.1 ± 1.55	26.8 ± 0.99	33.0 ± 0.95
Total Longevity (day)	24.4 ± 0.71	26.6 ± 1.12	40.3 ± 1.35	21.5 ± 1.68	34.0 ± 1.09	41.4 ± 1.35
