# Supplementary material for: Performances of survival, feeding behavior, and gene expression in aphids reveal their different fitness to host alteration
Source: Sci Rep. 2016 Jan 13;6:19344. doi: 10.1038/srep19344 (PMC4725932; doi:10.1038/srep19344)
Supplement: Supplementary Information [file srep19344-s1.pdf]

# Performances of survival, feeding behavior, and gene expression in aphids reveal their different fitness to host alteration

Hong Lu[a, b #], Pengcheng Yang[c #], Yongyu Xu[b #], Lan Luo[a], Junjie Zhu[a], Na Cui[a], Le Kang[* a], Feng Cui[* a]

[a] State Key Laboratory of Integrated Management of Pest Insects & Rodents, Institute of Zoology, Chinese Academy of Sciences, Beijing, China.

[b] Plant Protection College, Shandong Agricultural University, Tai'an, Shandong, China.

[c] Beijing Institutes of Life Science, Chinese Academy of Sciences, Beijing, China.

[#] These authors contribute equally to this work.

[*] Corresponding author: Feng Cui, State Key Laboratory of Integrated Management of Pest Insects & Rodents, Institute of Zoology, Chinese Academy of Sciences, Beijing, 100101 China. Tel: 86-10-64807218. Email: cuif@ioz.ac.cn. Le Kang, State Key Laboratory of Integrated Management of Pest Insects & Rodents, Institute of Zoology, Chinese Academy of Sciences, Beijing, 100101 China. Tel: 86-10-64807219. Email: lkang@ioz.ac.cn.

## Supporting Information

Data S1. List of salivary gland-expressed genes of the pea aphid.

Data S2. The enriched GO molecular functions (Level 3) for all salivary

gland-expressed genes and the genes putatively encoding secretory proteins of pea aphids.

Data S3. List of differently expressed genes in the YYC clone after feeding on *Vicia villosa*, *Medicago truncatula*, or *Medicago sativa* for 5 h. The genes that varied in aphids for specific host plant are in bold.

Data S4. Commonly upregulated and downregulated genes when the YYC clone was short-term shifted to *Vicia villosa*, *Medicago truncatula*, or *Medicago sativa*, and after long-term acclimated on *V. villosa* or *M. truncatula*.

Data S5. List of differently expressed genes in the *Medicago truncatula* and *Vicia villosa* colony compared with the YYC clone. The genes that varied in a specific colony are in bold.
