# Supplementary material for: Performances of survival, feeding behavior, and gene expression in aphids reveal their different fitness to host alteration
Source: Sci Rep. 2016 Jan 13;6:19344. doi: 10.1038/srep19344 (PMC4725932; doi:10.1038/srep19344)

Performances of survival, feeding behavior, and gene expression in aphids reveal their different fitness to host alteration

Hong Lu, Pengcheng Yang, Yongyu Xu, Lan Luo, Junjie Zhu,Na Cui, Le Kang, Feng Cui

Data S2. The enriched GO molecular functions (Level 3) for all salivary gland-expressed genes and the genes putatively encoding secretory proteins of pea aphids.

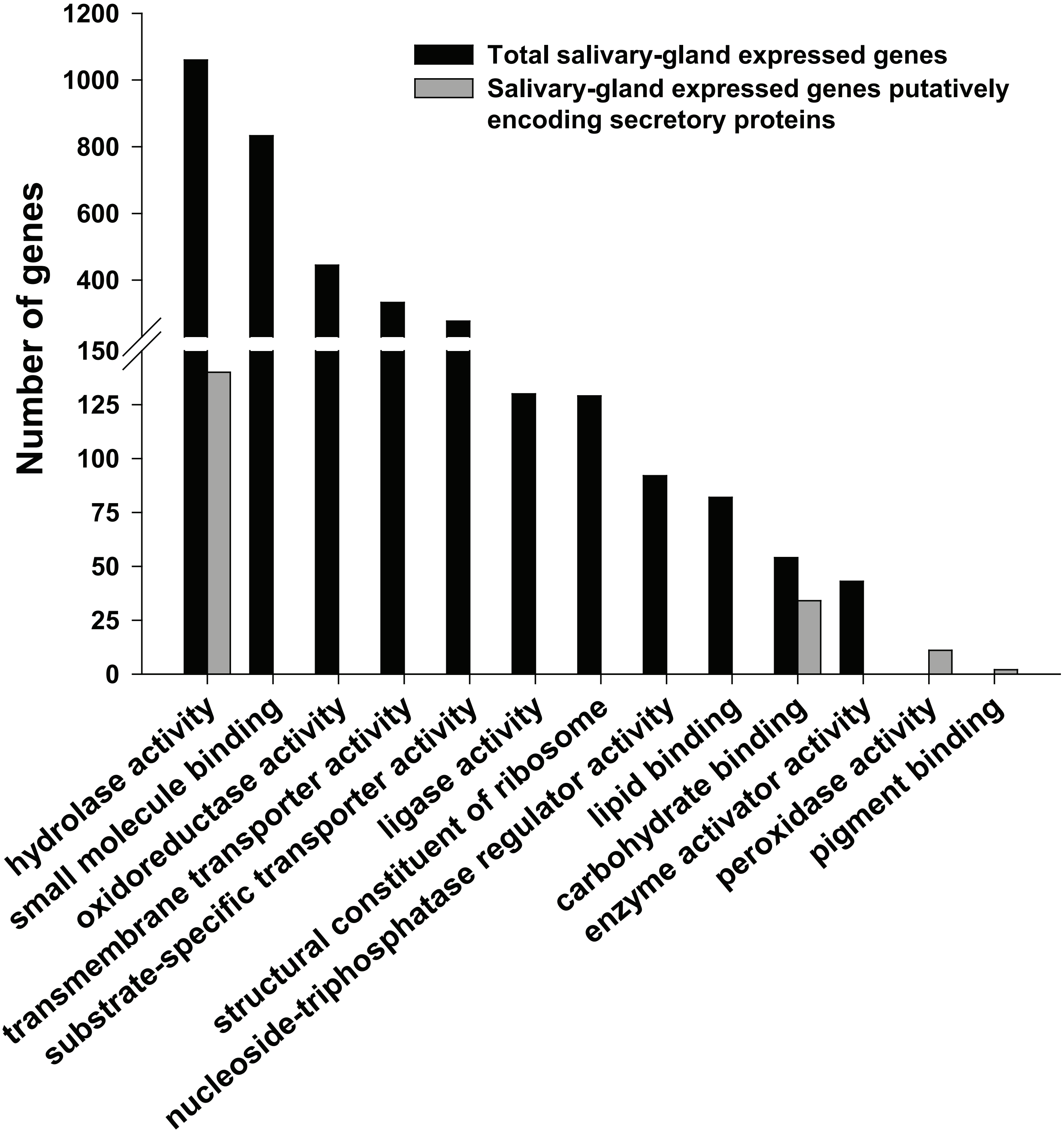

Supplement: Supplementary Dataset 1 [file srep19344-s2.pdf]
