# Supplementary material for: Performances of survival, feeding behavior, and gene expression in aphids reveal their different fitness to host alteration
Source: Sci Rep. 2016 Jan 13;6:19344. doi: 10.1038/srep19344 (PMC4725932; doi:10.1038/srep19344)
Supplement: Supplementary Dataset 2 [file srep19344-s3.pdf]

Performances of survival, feeding behavior, and gene expression in aphids reveal their different fitness to host alteration

Hong Lu, Pengcheng Yang, Yongyu Xu, Lan Luo, Junjie Zhu, Na Cui, Le Kang, Feng Cui

Data S4. Commonly upregulated and downregulated genes when the YYC clone was short-term shifted to *Vicia villosa*, *Medicago truncatula*, or *Medicago sativa*, and after long-term acclimated on *V. villosa* or *M. truncatula*.

**Annotation**	**Gene ID**
**short-term shift**	
**co-downregulated**	
heat shock proteins	ACYPI002010, ACYPI21638, ACYPI21636, ACYPI004698, ACYPI009117
52 KDa repressor of the inhibitor of the protein kinase	ACYPI34603
unknown proteins	ACYPI22179, ACYPI51701
**long-term acclimation**	
**co-upregulated**	
alpha-(1,6)-fucosyltransferase	ACYPI24917
acyl-CoA-binding protein	ACYPI001471
calpain-7	ACYPI008750
transcription elongation factor 1	ACYPI27126
syntaxin-18	ACYPI30696
unknown proteins	ACYPI005818, ACYPI27259, ACYPI56347, ACYPI003041, ACYPI23163, ACYPI21823, ACYPI41715, ACYPI30075, ACYPI30076, ACYPI30077, ACYPI45829, ACYPI52855, ACYPI54675, ACYPI22815, ACYPI41716, ACYPI22179, ACYPI51492, ACYPI45828, ACYPI52854, ACYPI52524, ACYPI45615, ACYPI45830, ACYPI40266, ACYPI23443, ACYPI20968, ACYPI46969, ACYPI43357, ACYPI20392, ACYPI35626, ACYPI21765, ACYPI42501, ACYPI38875, ACYPI39384, ACYPI26742, ACYPI002861, ACYPI45859, ACYPI47628, ACYPI36246, ACYPI23754, ACYPI31429, ACYPI40702, ACYPI38521, ACYPI26814, ACYPI35193, ACYPI21720
**co-downregulated**	
takeout protein	ACYPI001035, ACYPI25522, ACYPI24182
RNA-binding protein 14	ACYPI005750
adhesive plaque matrix protein	ACYPI007563
suppressor protein SRP40	ACYPI001839

apolipophorin-3	ACYPI36683
miple protein	ACYPI37161
titin –like	ACYPI36932, ACYPI008945
bolA-like protein 2	ACYPI001854
probable basic-leucine zipper transcription factor Q	ACYPI009169
unknown proteins	ACYPI005108, ACYPI010002, ACYPI007459, ACYPI005249, ACYPI44068, ACYPI44495, ACYPI27628, ACYPI54512, ACYPI000149, ACYPI51701
